# Management of complex ventral hernias: results of an international survey

**DOI:** 10.1093/bjsopen/zraa057

**Published:** 2021-02-11

**Authors:** L Knaapen, O Buyne, N Slater, B Matthews, H Goor, C Rosman

**Affiliations:** 1 Department of Surgery, Radboud University Medical Centre, Nijmegen, the Netherlands; 2 Department of Plastic and Reconstructive Surgery, Radboud University Medical Centre, Nijmegen, the Netherlands; 3 Department of Surgery, Carolinas Medical Center, Charlotte, North Carolina, USA

## Abstract

**Background:**

The surgical treatment of patients with complex ventral hernias is challenging. The aim of this study was to present an international overview of expert opinions on current practice.

**Methods:**

A survey questionnaire was designed to investigate preoperative risk management, surgical approach and mesh choice in patients undergoing complex hernias repair, and treatment strategies for infected meshes. Geographical location of practice, experience and annual volumes of the surgeons were compared.

**Results:**

Of 408 surgeons, 234 (57.4 per cent) were practising in the USA, 116 (28.4 per cent) in Europe, and 58 (14.2 per cent) in other countries. Some 412 of 418 surgeons (98.6 per cent) performed open repair and 322 of 416 (77.4 per cent) performed laparoscopic repair. Most recommended preoperative work-up/lifestyle changes such as smoking cessation (319 of 398, 80.2 per cent) and weight loss (254 of 399, 63.7 per cent), but the consequences of these strategies varied. American surgeons and less experienced surgeons were stricter. Antibiotics were given at least 1 h before surgery by 295 of 414 respondents (71.3 per cent). Synthetic and biological meshes were used equally in contaminated primary hernia repair, whereas for recurrent hernia repair synthetic mesh was used in a clean environment and biological or no mesh in a contaminated environment. American surgeons and surgeons with less experience preferred biological mesh in contaminated environments significantly more often. Percutaneous drainage and antibiotics were the first steps recommended in treating mesh infection. In the presence of sepsis, most surgeons favoured synthetic mesh explantation and further repair with biological mesh.

**Conclusion:**

There remains a paucity of good-quality evidence in dealing with these hernias, leading to variations in management. Patient optimization and issues related to mesh choice and infections require well designed prospective studies.

## Teaser

This survey provided a good overview of current practice among difficult subjects in ventral hernia repair on which limited guidelines exist in two continents (Europe and USA). Based on the findings, the authors recommend that more attention should be paid to preoperative optimization, specifically preoperative diabetic control and adequate antibiotic prophylaxis. Prehabilitation programmes should be implemented together with patient education on the importance of risk reduction and the impact of complications. Reaching goals set to optimize patients before hernia surgery should be a quality control parameter. The use of biological mesh in ventral hernia repair should be appraised critically. To improve adherence to guidelines and protocol decision tools, guideline training, easy ways of data-sharing, feedback loops and apps may be helpful.

## Resumen

**Antecedentes:**

El tratamiento quirúrgico de las eventraciones complejas es un desafío. El objetivo de este estudio fue presentar la opinión de expertos internacionales sobre la práctica clínica actual.

**Métodos:**

Se diseñó una encuesta para analizar la forma de afrontar el riesgo preoperatorio, la elección del abordaje quirúrgico y de la malla en pacientes a los que se va a realizar una reparación compleja de una eventración, así como la estrategia de tratamiento de una malla infectada. Se comparó la procedencia, la experiencia y el volumen anual de los cirujanos.

**Resultados:**

De los 408 cirujanos que participaron en el estudio, 205/408 (58%) ejercían en los EE.UU., 116/408 (28%) en Europa y 58/408 (14%) en otros países. Un 99% (412/418) prefería la reparación abierta y un 77% (322/416) la reparación laparoscópica. Los cambios de estilo de vida más recomendados en el preoperatorio fueron dejar de fumar (319/398; 80%) y perder peso (254/399, 64%), aunque los resultados de esta recomendación fueron dispares. Los cirujanos estadounidenses y los cirujanos menos experimentados fueron más estrictos. Administraban antibióticos al menos una hora antes de la operación 295/414 (71%) de los encuestados. En la reparación de hernias contaminadas, se utilizaron mallas sintéticas y biológicas por igual, mientras que para la reparación de hernias recidivadas se utilizó una malla sintética en entornos limpios y mallas biológicas o sin malla en entornos contaminados. Los cirujanos estadounidenses y los cirujanos con menos experiencia a menudo preferían la malla biológica en entornos contaminados. Los primeros gestos recomendados para tratar la infección de la malla fueron el drenaje percutáneo y los antibióticos. En presencia de sepsis, la mayoría de los cirujanos aconsejaron la extracción de la malla sintética y la reparación posterior con malla biológica.

**Conclusión:**

Existen pocos trabajos de calidad acerca del tratamiento de estas hernias, lo que lleva a una gran diversidad en la forma de afrontarlas. Se precisan estudios prospectivos bien diseñados que aborden la optimización del paciente, la elección de la malla y el tratamiento de las infecciones.

## Introduction

The surgical treatment of patients with complex ventral hernias is challenging. Complexity is defined according to the criteria proposed by Slater *et al*.^1^, and depends on the variables of size and location, contamination and soft tissue condition, patient history and risk factors, and the clinical scenario. Preoperative risk management, surgical technique, choice of mesh for contaminated wound conditions, and treatment of an infected mesh are the main topics addressed by guidelines designed to establish best clinical practice^2–4^.

Preoperative risk management includes patient optimization measures such as smoking cessation, controlling blood glucose in patients with diabetes, and weight reduction in obese patients to reduce complications^5^. Choice of mesh and appropriate surgical technique, especially in contaminated or infected operative fields, are particularly controversial as no well designed RCTs have proven superiority of one mesh type over another, or provided clear advantages for a particular surgical approach. Treatment of postoperative mesh infection has similarly been based on empirical findings. Rates of surgical-site infection and mesh infection range from 6 to 10 per cent for clean repair and up to 36 per cent in (potentially) contaminated fields^6^. Mesh infection in clinical practice seems to be widely treated by long-term antibiotic regimens with or without percutaneous drainage, or by mesh removal, again without strong evidence for superiority of a specific approach^7^.

In the absence of scientific evidence, expert opinion covering these issues was sought by developing a survey questionnaire in a collaboration between the USA and the Netherlands. Differences in geographical location of practice (USA *versus* Europe), experience and annual volume of the operating surgeon were evaluated along with results of responses compared with recommendations in clinical guidelines^2–4^.

## Methods

Invitations for the English web-based survey were sent to members of the American Hernia Society (AHS). A reminder was sent after 6 weeks. In addition, European hernia surgeons attending the first world conference on abdominal wall hernia surgery in Milan, Italy, in 2015 who were not members of the AHS were also offered the opportunity to take part.

### The survey

The survey consisted of 34 questions: 10 demographic questions, 9 knowledge questions, and 15 case vignettes (*Appendix S1*), designed for completion in around 20 min.

Information on participants included sex, age, geographical location of practice, experience in hernia surgery, and type of practice. Experience was considered to be ‘expert level’ when more than 50 ventral hernia procedures were performed annually. Preoperative risk management parameters were smoking cessation, weight loss regimen, and blood glucose control.

Knowledge in relation to current literature was based on nine multiple choice questions related to ventral hernia repair, knowledge of surgical wound classification, risk factors for ventral hernia recurrence, and surgical technique of hernia repair. Questions were considered ‘correct’ when the answer corresponded with recommendations provided by current guidelines^2–4^. Case vignettes explored surgical decision-making in a co-morbid patient, in clean or contaminated hernia repairs, treatment of mesh infection, and handling unplanned enterotomy.

### Statistical analysis

Data were anonymized and analysed using SPSS^®^ version 23 (IBM, Armonk, NY, USA). Answers were described as counts and percentages for categorical variables. Additionally, three different demographics within the preoperative risk management results, knowledge questions and the case vignettes were examined separately: geographical location of practice (USA *versus* Europe), experience of the surgeon (little (15 years or less) *versus* high (more than 15 years)) and annual volume of the operating surgeon (low (fewer than 50 per year) *versus* high (more than 50 per year)). The Freeman–Halton test was used to compare the results of the different case vignettes. *P* <0.050 was considered statistically significant for all tests.

## Results

### General characteristics of respondents

Questionnaires were returned by 417 surgeons, representing about 25 per cent of eligible members of hernia societies approached. Not all questions from the questionnaire were answered by all respondents, so the denominator used to calculate percentages varied. Responder demographics are listed in *Table 1*. Of 408 surgeons, 234 (57.4 per cent) were practising hernia surgery in the USA, 116 (28.4 per cent) in Europe, and 58 (14.2 per cent) in other countries. Some 412 of 418 surgeons (98.6 per cent) performed open repair, 105 of 409 (25.7 per cent) at the expert level. Laparoscopic repair was performed by 322 of 416 responders (77.4 per cent), of whom 69 of 320 (21.6 per cent) were at the expert level.

**Table 1:** Demographics of responders

	No. of respondents
**Age (years)**	*n* = 417
<30	6 (1.4)
31–35	23 (5.5)
36–45	88 (21.1)
46–55	153 (36.7)
56–65	110 (26.4)
>65	37 (8.9)
**Sex**	*n* = 416
M	381 (91.6)
F	35 (8.4)
**Time in practice (years)**	*n* = 417
<5	28 (6.7)
5–10	48 (11.5)
10–15	66 (15.8)
15–20	59 (14.1)
>20	216 (51.8)
**Geographical location of practice**	*n* = 408
Europe	116 (28.4)
USA	234 (57.4)
Other^*^	58 (14.2)
**Open ventral hernia repair**	*n* = 418
Yes	412 (98.6)
No	6 (1.4)
**Annual volume of open repair**	*n* = 409
<25	150 (36.7)
25–50	154 (37.7)
50–100	82 (20.0)
>100	23 (5.6)
**Laparoscopic ventral hernia repair**	*n* = 416
Yes	322 (77.4)
No	94 (22.6)
**Annual volume of laparoscopic repair**	*n* = 320
<25	152 (47.5)
25–50	99 (30.9)
50–100	48 (15.0)
>100	21 (6.6)

Values in parentheses are percentages/

* Africa (not specified), Argentina, Australia, Brazil, Chile, China, Colombia, Egypt, Estonia, Guatemala, India, Iraq, Japan, Libya, Macedonia, Mexico, Montenegro, Saudi Arabia, Singapore, Slovenia, Georgia, Venezuela.

### Preoperative risk management

Views concerning preoperative risk management are shown in *Table 2*.

**Table 2:** Preoperative optimization/lifestyle factors

Smoking cessation (*n*=398)		Diabetes control (HbA1c) (*n*=392)		Weight loss regimen (BMI >40 kg/m^2^) (*n*=399)	
No	79 (19.8)	No	227 (57.9)	No	145 (36.3)
Yes	319 (80.2)	Yes	165 (42.1)	Yes	254 (63.7)
1–2 weeks	63 (19.7)	<6.0%	58 (35.2)	<5–10%	83 (32.7)
1 month^*^	174 (54.5)	6.1–7.0%^*^	75 (45.5)	11–20%^*^	54 (21.3)
3 months	57 (17.9)	7.1–8.0%	22 (13.3)	>20%	11 (4.3)
No preference	23 (7.2)	>8.0%	1 (0.6)	No preference	46 (18.1)
		No preference	8 (4.8)	Referral for bariatric surgery	59 (23.2)

Values in parentheses are percentages.

* Recommendation in guidelines.

Some 319 of 398 surgeons (80.2 per cent) agreed on the benefit of smoking cessation. Fifty-one of 396 surgeons (12.9 per cent) checked urine for nicotine and metabolites before surgery, of whom 49 of 50 (98 per cent) demanded negative results. A total of 254 of 399 surgeons (63.7 per cent) recommended a weight loss regimen in patients with a high BMI (above 40 kg/m^2^) before surgery. Ninety-one of 146 surgeons (62.3 per cent) were willing to proceed to repair even if the target weight loss had not been reached. Some 165 of 392 surgeons (42.1 per cent) checked target haemoglobin (Hb) A1c to improve diabetes control before surgery; 68 of 156 surgeons (43.6 per cent) would operate on patients who did not reach the target HbA1c.

Antibiotics at least 1 h before surgery were recommended by 295 of 414 (71.3 per cent), of whom 80 (19.3 per cent) administered further antibiotics over the next 24 h, whereas 26 (6.3 per cent) continued for more than 24 h. Only 13 of 414 (3.1 per cent) did not use prophylactic antibiotics for ventral incisional hernia repair.

#### *American* versus *European surgeons*

Compared with European surgeons, significantly more American surgeons wanted patients to stop smoking (199 of 224 (88.8 per cent) *versus* 69 of 110 (62.7 per cent) respectively; *P* < 0.001), checked urine for nicotine (47 of 223 (21.1 per cent) *versus* 3 of 108 (2.8 per cent); *P* = 0.001) and determined HbA1c before surgery (101 of 218 (46.3 per cent) *versus* 31 of 110 (28.2 per cent); *P* = 0.006). No difference was found for weight loss regimens (149 of 225 (66.2 per cent) *versus* 64 of 110 (58.2 per cent); *P* = 0.199) or requirement of negative test results before operation (smoking: 45 of 46 (98 per cent) *versus* 3 of 3 (100 per cent), *P* = 0.796; weight loss: 50 of 78 (64 per cent) *versus* 27 of 42 (64 per cent), *P* = 0.757; HbA1c: 44 of 99 (44 per cent) *versus* 11 of 25 (44 per cent), *P* = 0.523).

#### *High* versus *low annual volume surgeons*

No difference was seen between surgeons with high or low annual volume concerning smoking cessation (145 of 189 (76.7 per cent) *versus* 90 of 110 (81.8 per cent) respectively; *P* = 0.380), weight loss regimen (122 of 189 (64.6 per cent) *versus* 77 of 111 (69.4 per cent); *P* = 0.448), checking HbA1c before surgery (83 of 185 (44.9 per cent) *versus* 50 of 109 (45.9 per cent); *P* = 0.904), or requirement for negative results before operation (smoking: 21 of 22 (95 per cent) *versus* 21 of 21 (100 per cent), *P* = 1.000; weight loss: 48 of 72 (67 per cent) *versus* 29 of 48 (60 per cent), *P* = 0.561; HbA1c: 37 of 80 (46 per cent) *versus* 19 of 49 (39 per cent), *P* = 0.466).

#### *Less experienced* versus *more experienced surgeons*

Less experienced surgeons were more likely to commence morbidly obese patients on a weight loss regimen than those with more experience (95 of 134 (70.9 per cent) *versus* 158 of 264 (59.8 per cent) respectively; *P* = 0.036) and checked urine for nicotine more often (31 of 132 (23.5 per cent) *versus* 20 of 263 (7.6 per cent); *P* < 0.001). No difference was found for smoking cessation (107 of 133 (80.5 per cent) *versus* 211 of 264 (79.9 per cent); *P* = 1.000), checking HbA1c before to surgery (53 of 129 (41.1 per cent) *versus* 111 of 262 (42.4 per cent); *P* = 0.828) or requirement for negative results before operation (smoking: 30 of 31 (97 per cent) *versus* 19 of 19 (100 per cent), *P* = 1.000; weight loss: 34 of 55 (62 per cent) *versus* 57 of 91 (63 per cent), *P* = 1.000; HbA1c: 22 of 51 (43 per cent) *versus* 46 of 104 (44.2 per cent), *P* = 1.000).

### Knowledge questions

The knowledge questions (Q1–Q9) with answers and references are shown in *Table 3*. Only the questions about surgical wound classification (Q3) and anatomical plane (Q6) produced consistent responses with 73.1 per cent and 80.1 per cent of respondents respectively giving the correct answer.

**Table 3:** Results of knowledge questions and answers of respondents

Survey question	Survey answers	No. of respondents
Q1: In your estimation, what percentage of all patients who experience ventral hernia recurrence undergo repeated ventral hernia repair? (*n* = 414)	a: 0–10%	63 (15.2)
	b: 11-20%^8^*	66 (15.9)
	c: 21–30%	60 (14.5)
	d: 31–40%	48 (11.6)
	e: 41–50%	38 (9.2)
	f: 51–60%	25 (6.0)
	g: 61–70%	33 (8.0)
	h: 71–80%	62 (15.0)
	i: 81–90%	11 (2.7)
	j: 91–100%	8 (1.9)
Q2: After primary/suture repair of a ventral incisional hernia that is >6 cm, most patients develop a recurrence within 10 years. What is the 10-year recurrence rate if an underlay mesh is used to reinforce the repair? (*n* = 403)	a: 16%^9^*	189 (46.9)
	b: 24%^9^*	124 (30.8)
	c: 32%	70 (17.4)
	d: 41%	20 (5.0)
Q3: A patient undergoes open ventral hernia repair for a recurrent, incarcerated ventral hernia. A section of small bowel requires resection due to dense adhesions to a prosthetic mesh. There is no inflammation or purulence around the mesh or bowel. What is the surgical wound classification? (*n* = 402)	a: Clean wound (class I)	12 (3.0)
	b: Clean-contaminated wound (class II)^*^	294 (73.1)
	c: Contaminated wound (class III)	94 (23.4)
	d: Dirty or infected wound (class IV)	2 (0.5)
Q4: Severity grading scales for postoperative complications are important for reporting outcomes. Consider this scenario: a patient develops a postoperative ileus after open ventral hernia repair and requires a short period of total parenteral nutrition (TPN). His recovery is otherwise uneventful. According to all the published grading systems for postoperative complications, what is the correct classification? (*n* = 388)	a: Grade 0/normal	16 (4.1)
	b: Grade 1/mild^10^*	185 (47.7)
	c: Grade 2/moderate	175 (45.1)
	d: Grade 3/Severe	12 (3.1)
Q5: According to the best available evidence, which of following co-morbidities is an independent risk factor for recurrence after ventral hernia repair regardless technique? (*n* = 388)	a: Abdominal aortic aneurysm	77 (19.8)
	b: Patient age^*^	17 (4.4)
	c: Steroid use^*^	98 (25.3)
	d: Prostatism	15 (3.9)
	e: Tobacco use^1^*	181 (46.6)
Q6: According to the Rives–Stoppa technique for incisional hernia repair, prosthetic mesh is placed in what anatomical plane? (*n* = 391)	a: Intraperitoneal	3 (0.8)
	b: Preperitoneal	73 (18.7)
	c: Retrorectus ^*^	313 (80.1)
	d: Prefascial	2 (0.5)
Q7: According to the component separation method for closure of the abdominal wall defects described by Ramirez *et al*., what is the typical maximum distance of bilateral advancement in the mid-abdomen? (*n* = 385)	a: 6 cm	30 (7.8)
	b: 10 cm	202 (52.5)
	c: 20 cm^11^*	141 (36.6)
	d: 24 cm	12 (3.1)
Q8: When a midline incision is closed with a continuous monofilament suture, what technique is associated with an increased rate of surgical site infection? (*n* = 381)	a: Suture length to wound length ratio of <4 : 1^12^*	199 (52.2)
	b: Suture length to wound length ratio of >4 : 1^12^*	182 (47.8)
Q9: Within 4 weeks of open ventral hernia repair, up to 21.9% of patients develop a surgical-site infection. What is the surgical-site infection rate within 4 weeks after laparoscopic ventral hernia repair? (*n* = 372)	a: 1.5%^13^*	127 (34.1)
	b: 2.8%	105 (28.2)
	c: 3.7%	72 (19.4)
	d: 5.1%	68 (18.3)

Values in parentheses are percentages.

* Correct answer.

#### *American* versus *European surgeons*

The majority of American surgeons reported a higher percentage of repeat ventral hernia repairs (Q1) compared with European surgeons. Forty-seven of 231 (20.3 per cent) chose answer h, in which the number of repeat hernia repairs was set at 71–80 per cent, *versus* 10 of 115 (8.7 per cent) of the European surgeons. In the latter group, answer a (0–10 per cent) was chosen more often: 29 of 115 (25.2 per cent) for European surgeons *versus* 16 of 231 (6.9 per cent) for American surgeons (*P* < 0.001). European surgeons considered ‘abdominal aortic aneurysm’ (answer a) more often to be an independent risk factor for recurrence (Q5) than American surgeons (30 of 108 (27.8 per cent) *versus* 35 of 216 (16.2 per cent) respectively; *P* = 0.001). American surgeons indicated ‘tobacco use’ (answer e) more often to be a risk factor than European surgeons (121 of 216 (56.0 per cent) *versus* 39 of 108 (36.1 per cent) respectively; *P* = 0.001). No differences between American and European surgeons were found for the other knowledge questions.

#### *High* versus *low annual volume surgeons*

The majority of high-volume surgeons reported a lower percentage of surgical-site infections (Q9) compared with low-volume surgeons. Fifty-three of 107 (49.5 per cent) chose answer a, in which the percentage was set at 1.5 per cent, compared with 52 of 183 (28.4 per cent) for low-volume surgeons (*P* = 0.004). No differences between surgeons with high or low annual volume were found for the other items.

#### *Less experienced* versus *more experienced surgeons*

Q2 on recurrence rate within 10 years was answered correctly more often by more experienced surgeons compared with less experienced surgeons (220 of 266 (82.7 per cent) *versus* 93 of 136 (68.4 per cent) respectively; *P* = 0.001). Conversely, less experienced surgeons answered the question about the typical maximum distance of bilateral advancement with the component separation method (Q7) correctly more often than more experienced surgeons (61 of 127 (48.0 per cent) *versus* 80 of 257 (31.1 per cent) respectively; *P* = 0.002). No differences between less and more experienced surgeons were found for the other items.

### Case vignettes

The case vignettes with all answers are shown in *Table 4*. The most frequently provided answers for the specific case vignette are discussed below.

**Table 4:** Results of case vignettes and answers of respondents. ^*^Correct answer

Case vignette	Survey answers	No. of respondents
**Surgical technique in co-morbid patient**		
A co-morbid patient (smoker, obese, diabetic, immunosuppressed, or with COPD) with an increased theoretical risk of surgical-site infection has a ventral incisional hernia >6 cm. There is no evidence of wound contamination or active infection. How would you approach this patient? (*n* = 408)	Primary closure without mesh	5 (1.2)
	Open repair with permanent synthetic mesh^*^	147 (36.0)
	Open repair with absorbable synthetic mesh	13 (3.2)
	Open repair with biological mesh	24 (5.9)
	Laparoscopic ventral hernia repair^*^	198 (48.5)
	Other	21 (5.1)
**Decision-making in clean and contaminated hernia repair**		
Which type of mesh do you prefer to use in the extraperitoneal space when reinforcing the open repair of a > 6-cm ventral incisional hernia at a CONTAMINATED surgical site? (*n* = 415)	Permanent synthetic mesh	144 (35.6)
	Absorbable synthetic mesh	36 (8.7)
	Biological mesh^*^	175 (42.2)
	Primary suture repair with possible autologous tissue transfer, no mesh reinforcement	60 (14.5)
If synthetic, which pore size? (*n* = 142)	Microporous mesh	15 (10.6)
	Macroporous mesh	108 (76.1)
	Mesh pore size is not a factor in my choice of mesh	19 (13.4)
If synthetic, which density? (*n* = 142)	Lightweight mesh	99 (69.7)
	Heavyweight mesh	22 (15.5)
	Mesh density is not a factor in my choice of mesh	21 (14.8)
The patient experiences a ventral hernia recurrence. If the surgical site is now CLEAN (NO LONGER CONTAMINATED), which type of mesh do you prefer to use in the extraperitoneal space when reinforcing the open repair of the >6-cm ventral hernia recurrence? (*n* = 413)	Permanent synthetic mesh^*^	344 (83.3)
	Absorbable synthetic mesh	15 (3.6)
	Biological mesh	40 (9.7)
	Primary suture repair with possible autologous tissue transfer, no mesh reinforcement	14 (3.4)
If synthetic, which pore size? (*n* = 341)	Microporous mesh	35 (10.3)
	Macroporous mesh^*^	255 (74.8)
	Mesh pore size is not a factor in my choice of mesh	51 (15.0)
If synthetic, which density? (*n* = 340)	Lightweight mesh^*^	237 (69.5)
	Heavyweight mesh	59 (17.3)
	Mesh density is not a factor in my choice of mesh	44 (12.9)
The patient experiences a ventral hernia recurrence. If the surgical site is STILL CONTAMINATED, which type of mesh do you prefer to use in the extraperitoneal space when reinforcing the open repair of the >6-cm ventral hernia recurrence? (*n* = 408)	Permanent synthetic mesh	37 (9.1)
	Absorbable synthetic mesh	44 (10.8)
	Biological mesh^*^	215 (52.7)
	Primary suture repair with possible autologous tissue transfer, no mesh reinforcement^*^	112 (27.5)
If synthetic, which pore size? (*n* = 37)	Microporous mesh	6 (16)
	Macroporous mesh	27 (73)
	Mesh pore size is not a factor in my choice of mesh	4 (11)
If synthetic, which density? (*n* = 36)	Lightweight mesh	25 (69)
	Heavyweight mesh	6 (17)
	Mesh density is not a factor in my choice of mesh	5 (14)
During an open ventral incisional hernia repair a 4-cm gap between the rectus muscles can be easily approximated. The patient has no co-morbidities, no history of wound infection, and no evidence of contamination. How would you proceed? (*n* = 397)	Bridge the defect with mesh	11 (2.8)
	Close the fascia primarily without mesh	24 (6.0)
	Reinforce the fascial closure with an intraperitoneal mesh	53 (13.4)
	Reinforce the fascial closure with a retrorectus mesh (Rives–Stoppa repair)^*^	256 (64.5)
	Reinforce the fascial closure with an onlay mesh	48 (12.1)
	Other	5 (1.3)
In the patient above, if a 7-cm gap between the rectus muscles cannot be approximated without undue tension during an open case, how would you perform the repair? (*n* = 394)	Bridge the defect with mesh	36 (9.1)
	Anterior component separation and close the fascia primarily without mesh	5 (1.3)
	Anterior component separation and reinforce the fascial closure with an intraperitoneal mesh	44 (11.2)
	Anterior component separation and reinforce the fascial closure with a retrorectus mesh (Rives–Stoppa repair)^*^	181 (45.9)
	Anterior component separation and reinforce the fascial closure with an onlay mesh	37 (9.4)
	Posterior component separation and reinforce the fascial closure with a retrorectus mesh^*^	76 (19.3)
	Other	15 (3.8)
How would you approach a patient with a nearby stoma and a ventral incisional hernia >6 cm? (*n* = 391)	Primary closure without mesh	9 (2.3)
	Open repair with permanent synthetic mesh^*^	120 (30.7)
	Open repair with absorbable synthetic mesh^*^	20 (5.1)
	Open repair with biological mesh^*^	82 (21.0)
	Laparoscopic ventral hernia repair^*^	154 (39.4)
	Other	6 (1.5)
**Mesh infection and unplanned enterotomy**		
Three weeks after open component separation and retrorectus placement of a biological mesh, a patient develops fever, leucocytosis, and a fluid collection adjacent to the mesh. The patient has no other obvious source of infection. How would you manage this patient? Begin empirical antibiotics and… (*n* = 403)	Observation	59 (14.6)
	CT-guided percutaneous drain^*^	250 (62.0)
	Laparoscopic exploration with drainage of fluid and complete mesh removal	12 (3.0)
	Open drainage of the fluid and completely excise the mesh	24 (6.0)
	Open drainage of the fluid and excise unincorporated mesh	48 (11.9)
	Open drainage of the fluid and leave the mesh in place	10 (2.5)
Three weeks after placement of a permanent barrier composite mesh during laparoscopic repair, a patient develops fever, leucocytosis, and a fluid collection between the mesh and abdominal wall. The patient has no other obvious source of infection. How would you manage this patient? Begin empirical antibiotics and… (*n* = 398)	Observation	54 (13.6)
	CT-guided percutaneous drain^*^	181 (45.5)
	Laparoscopic exploration with drainage of fluid and complete mesh removal	37 (9.3)
	Open drainage of the fluid and completely excise the mesh	62 (15.6)
	Open drainage of the fluid and excise unincorporated mesh	18 (4.5)
	Open drainage of the fluid and leave the mesh in place	34 (8.5)
	Other	12 (3.0)
In the scenario above, fluid culture from a CT-guided percutaneous drain reveals MRSA. How would you manage this patient? (*n* = 179)	Continue antibiotics and leave the CT-guided drain in place until resolution of the abscess^*^	82 (45.8)
	Open exploration, mesh removal, and primary hernia repair	24 (13.4)
	Open exploration, mesh explantation, and repair with biological mesh	40 (22.3)
	Open exploration, mesh removal, and repair with absorbable synthetic mesh	11 (6.1)
	Laparoscopic removal of the mesh	19 (10.6)
	Other	3 (1.7)
How would you approach a patient with a recurrent parastomal hernia and an infected synthetic mesh without systemic sepsis? Mesh removal and… (*n* = 399)	Primary closure without mesh	91 (22.8)
	Repair with permanent synthetic mesh	21 (5.3)
	Repair with absorbable synthetic mesh	31 (7.8)
	Repair with biological mesh	217 (54.4)
	Other	39 (9.8)
How would you approach a patient with a history of MRSA wound infection and a ventral incisional hernia >6 cm? There is no active infection at this time. (*n* = 395)	Primary closure without mesh	18 (4.6)
	Open repair with permanent synthetic mesh^*^	117 (29.6)
	Open repair with absorbable synthetic mesh	18 (4.6)
	Open repair with biological mesh	76 (19.2)
	Laparoscopic ventral hernia repair^*^	150 (38.0)
	Other	16 (4.1)
Three weeks after open component separation and retrorectus placement of a macroporous mesh, a patient develops fever, leucocytosis, and a fluid collection adjacent to the mesh. The patient has no other obvious source of infection. How would you manage this patient? Begin empirical antibiotics and… (*n* = 394)	Observation	59 (15.0)
	Place a CT-guided percutaneous drain^*^	225 (57.1)
	Open exploration with fluid drainage and complete mesh removal	25 (6.3)
	Open exploration with fluid drainage and excision of unincorporated mesh	18 (4.6)
	Open exploration with fluid drainage, leaving the mesh in place	59 (15.0)
	Other	8 (2.0)
If the wound was opened in the scenario above, how would you address the skin closure if there is minimal cellulitis and it can be easily approximated? (*n* = 102)	Primary closure of the skin	5 (4.9)
	Loose closure of the skin with draining wicks	16 (15.7)
	Pack the wound with gauze and allow closure by secondary intention	9 (8.8)
	Apply a negative-pressure dressing and allow closure by secondary intention^*^	54 (52.9)
	Delayed primary closure	13 (12.7)
	Other	5 (4.9)
In the event of an unplanned enterotomy with minimal spillage during elective laparoscopic ventral incisional hernia repair, how would you typically repair the enterotomy? (*n* = 375)	Repair the enterotomy laparoscopically^*^	237 (63.2)
	Convert to open and repair the enterotomy	129 (34.4)
	Other	9 (2.4)

Values in parentheses are percentages.

* Correct answer. COPD, chronic obstructive pulmonary disease; MRSA, methicillin-resistant *Staphylococcus aureus*.

#### Surgical technique in co-morbid patient

In the case of a co-morbid patient (such as smoker, obese, diabetic, immunosuppressed or with chronic obstructive pulmonary disease), 184 (45.1 per cent) of the 408 respondents preferred open repair and 198 (48.5 per cent) preferred laparoscopic repair. Permanent synthetic mesh was used most often in open repairs (147 of 184, 79.9 per cent). For laparoscopic ventral hernia repair, the type of mesh was not specified.

#### Decision-making in clean and contaminated hernia repair

In contaminated primary hernia repair, synthetic mesh (180 of 415, 43.4 per cent) was used as often as biological mesh (175 of 415, 42.2 per cent). Primary suture repair was performed by 60 (14.5 per cent) of the 415 respondents. In case of a contaminated recurrent hernia repair, most surgeons preferred a biological (215 of 408, 52.7 per cent) over a synthetic (81 of 408, 19.9 per cent) mesh. Primary suture repair was performed by 112 (27.5 per cent) of the 408 respondents.

In clean recurrent hernia repair, 359 of 413 respondents (86.9 per cent) preferred a synthetic over a biological mesh (40 of 413, 9.7 per cent). Primary suture repair was performed by 14 (3.4 per cent) of the 413 respondents. Most preferred a permanent (344 of 413, 83.3 per cent), macroporous (255 of 341, 74.8 per cent) or lightweight (237 of 341, 69.5 per cent) mesh in clean recurrent hernia repair.

In patients with a defect larger than 6 cm and a stoma in close proximity, most respondents chose either an open repair with permanent synthetic mesh (120 of 391, 30.7 per cent), open repair with biological mesh (82 of 391, 21.0 per cent) or laparoscopic ventral hernia repair with the type of mesh not specified (154 of 391, 39.4 per cent).

For a non-co-morbid patient during an open clean ventral incisional hernia repair with a 4-cm gap between the rectus muscles that could easily be approximated, most respondents reinforced the fascial closure with a retrorectus mesh (256 of 397, 64.5 per cent). If the gap was 7 cm and could not be approximated during open repair, either anterior component separation (181 of 394, 45.9 per cent) or posterior component separation (76 of 394, 19.3 per cent) was added to the retrorectus reinforcement.

#### Mesh infection

In the case vignettes on mesh infection, the first step in treatment was to start empirical antibiotics. In mesh infection occurring after an open component separation and retrorectus placement of a biological mesh, 250 (62.0 per cent) of 403 surgeons would drain the fluid percutaneously, whereas 24 (6.0 per cent) preferred open drainage of the fluid and complete excision the mesh. In case of mesh infection after laparoscopic application of a permanent barrier composite mesh, 181 of 398 (45.5 per cent) would drain the fluid percutaneously and 62 of 398 (15.6 per cent) preferred open drainage of the fluid and complete excision the mesh. After open component separation and retrorectus placement of a macroporous synthetic mesh, 225 of 394 surgeons (57.1 per cent) would drain the fluid percutaneously, and 25 of 394 (6.3 per cent) preferred open exploration with fluid drainage and complete mesh removal.

If fluid culture from the drain revealed methicillin-resistant *Staphylcoccus aureus* (MRSA) infection, 82 of 179 respondents (45.8 per cent) continued antibiotics and left the mesh with the CT-guided drain in place until resolution of infection, and 94 of 179 (52.5 per cent) explanted the mesh.

For a patient with a history of MRSA wound infection (no current active infection) and a ventral incisional hernia greater than 6 cm, 150 of 395 respondents (38.0 per cent) chose laparoscopic repair (mesh not specified), 117 of 395 (29.6 per cent) chose open repair with permanent synthetic mesh, and 76 of 395 (19.2 per cent) selected open repair with biological mesh.

In a patient with a recurrent parastomal hernia and an infected synthetic mesh without systemic sepsis, more than half of the responders would explant the mesh followed by repair with a biological mesh (217 of 399, 54.4 per cent) or primarily close the parastomal hernia without mesh (91 of 399, 22.8 per cent).

#### Unplanned enterotomy

In the event of an unplanned enterotomy with minimal spillage during elective laparoscopic ventral incisional hernia repair, 237 of 375 respondents (63.2 per cent) would repair the enterotomy laparoscopically. In this situation, 148 (39.5 per cent) of 375 surgeons would then delay the hernia repair, 122 (32.5 per cent) would proceed laparoscopically, 94 (25.1 per cent) would convert to an open procedure, and 11 (2.9 per cent) did not further specify their hernia repair. If laparoscopy was continued, 69 of 120 (57.5 per cent) preferred a permanent synthetic mesh repair, 16 of 120 (13.3 per cent) preferred absorbable synthetic mesh repair, and 35 of 120 (29.2 per cent) preferred a biological repair. When converting to open ventral hernia repair, 28 of 91 (31 per cent) preferred permanent synthetic mesh repair, 11 of 91 (12 per cent) preferred absorbable synthetic repair, and 52 of 91 (57 per cent) preferred a biological mesh repair. There was no agreement about the length of delay (3 days: 32 of 146, 22 per cent; 2 weeks: 17 of 146, 11.6 per cent; 1 month: 15 of 146, 10.3 per cent; 6 weeks: 43 of 146, 29.5 per cent; 3 months: 32 of 146, 21.9 per cent; 6 months: 7 of 146, 4.8 per cent).

#### *American* versus *European surgeons*

American surgeons significantly preferred laparoscopic over ventral hernia repair in patients with co-morbidity compared with European surgeons (118 of 229 (51.5 per cent) *versus* 43 of 114 (37.7 per cent) respectively; *P* = 0.004), and were more likely to use biological mesh in contaminated primary (129 of 232 (55.6 per cent) *versus* 33 of 115 (28.7 per cent); *P* < 0.001) and recurrent (138 of 226 (61.1 per cent) *versus* 51 of 115 (44.3 per cent); *P* = 0.008) hernia repair. Although both American and European surgeons preferred permanent synthetic mesh (181 of 231 (78.4 per cent) *versus* 107 of 115 (93.0 per cent) in clean recurrent hernia repair, significantly more American surgeons chose biological mesh (30 of 231 (13.0 per cent) *versus* 5 of 115 (4.3 per cent) (*P* = 0.005).

If a patient had a ventral incisional hernia of more than 6 cm and a stoma in close proximity, European surgeons preferred open repair with permanent synthetic mesh (44 of 107 (41.1 per cent) *versus* 56 of 219 (25.6 per cent) for American surgeons; *P* = 0.030), whereas most American surgeons chose open repair with biological mesh (53 of 219 (24.2 per cent) *versus* 19 of 107 (17.8 per cent)) or laparoscopic repair (mesh not specified) (93 of 219 (42.5 per cent) *versus* 35 of 107 (32.7 per cent)) (*P* = 0.030).

For mesh infection after open component separation and retrorectus placement of a biological mesh, European surgeons preferred conservative management (with antibiotics) (29 of 111 (26.1 per cent) *versus* 21 of 227 (9.3 per cent) for American surgeons), whereas American surgeons more often intervened by draining the fluid percutaneously (163 of 227 (71.8 per cent) *versus* 50 of 111 (45.0 per cent) for European surgeons) (*P* < 0.001). A mesh infection after open component separation and retrorectus placement of a macroporous synthetic mesh was more likely to be treated conservatively by European surgeons (with antibiotics) (25 of 110 (22.7 per cent) *versus* 23 of 219 (10.5 per cent) for American surgeons) or open exploration with fluid drainage, leaving the mesh in place (22 of 110 (20.0 per cent) *versus* 23 of 219 (10.5 per cent) respectively), whereas American surgeons preferred draining the fluid percutaneously (136 of 219 (62.1 per cent) *versus* 56 of 110 (50.9 per cent) for European surgeons) (*P* = 0.001). With mesh infection after laparoscopic application of a permanent barrier composite mesh more European surgeons preferred conservative management (with antibiotics) (23 of 109 (21.1 per cent) *versus* 22 of 226 (9.7 per cent) of American surgeons), whereas American surgeons more often intervened by open drainage of the fluid and complete excision of the mesh (46 of 226 (20.4 per cent) *versus* 8 of 109 (7.3 per cent) of European surgeons) (*P* = 0.002).

If a patient had a recurrent parastomal hernia with an infected synthetic mesh (without systemic sepsis), American surgeons were more inclined to repair the parastomal hernia with a biological mesh (138 of 225 (61.3 per cent) *versus* 50 of 109 (45.9 per cent) of European surgeons; *P*<0.001) after removal of the infected mesh. European surgeons were more likely to close the parastomal hernia without mesh.

In the event of an unplanned enterotomy with minimal spillage during elective laparoscopic repair, more European surgeons would repair the enterotomy laparoscopically than American surgeons (79 of 102 (77.5 per cent) *versus* 120 of 214 (56.1 per cent) respectively; *P* = 0.001).

#### *Less experienced* versus *more experienced surgeons*

In a patient with no co-morbidity and a hernia with a 7-cm gap that could not be approximated during open repair, surgeons with less experience more often chose the posterior component separation technique (36 of 130 (27.7 per cent) *versus* 39 of 263 (14.8 per cent)), compared with experienced surgeons who more often preferred the anterior component separation technique (52 of 130 (40.0 per cent) *versus* 129 of 263 (49.0 per cent) respectively) (*P* = 0.050). Both techniques used reinforcement with a retrorectal mesh.

In contaminated primary hernia repair, surgeons with little experience chose a biological mesh (72 of 142 (50.7 per cent) *versus* 103 of 272 (37.9 per cent)) significantly more often than experienced surgeons, who more often chose a permanent synthetic mesh (41 of 142 (28.9 per cent) *versus* 102 of 272 (37.5 per cent)) (*P* = 0.003).

In the case vignettes on mesh infection, both groups chose percutaneous drainage as the first step after antibiotics. If this failed, experienced surgeons preferred open drainage of the fluid and leaving the mesh in place as the next step (41 of 266, 15.4 per cent), compared with less experienced surgeons (7 of 136, 5.1 per cent) (*P* = 0.030), who chose to continue with conservative therapy.

#### *High* versus *low annual volume*

For mesh infection, high-volume surgeons preferred percutaneous drainage significantly more often than low-volume surgeons (89 of 114 (78.1 per cent) *versus* 115 of 193 (59.6 per cent); *P* = 0.040), whereas surgeons with a low annual volume more often preferred conservative management (with antibiotics) (30 of 193 (15.5 per cent) *versus* 11 of 114 (9.6 per cent) of high-volume surgeons) or open drainage, leaving the mesh in place (23 of 193 (11.9 per cent) *versus* 8 of 114 (7.0 per cent) respectively).

In case of a mesh abscess after open component separation and retrorectus placement of a macroporous synthetic mesh, surgeons with a low annual volume more often preferred conservative management (with antibiotics) (34 of 186 (18.3 per cent) *versus* 11 of 109 (10.1 per cent) for high-volume surgeons) and open exploration with fluid drainage with complete mesh removal (19 of 186 (10.2 per cent) *versus* 1 of 109 (0.9 per cent) respectively), and those with a high annual volume more often preferred percutaneous drainage (78 of 109 (71.6 per cent) *versus* 94 of 186 (50.5 per cent) for low-volume surgeons) (*P* = 0.001).

## Discussion

This article provides an international overview of self-reported practice on different topics in ventral hernia repair in relation to practice location, experience of the operating surgeon, and annual volume of the operating surgeon. The striking feature was the extent of variation for many responses.

Preoperative optimization and lifestyle measures, related to smoking cessation (more than 4 weeks before surgery), maintaining blood glucose (HbA1c below 7 per cent) in patients with diabetes, and weight-loss regimens (to attain BMI below 30 kg/m^2^), have all been recommended by European and American ventral hernia working groups^2^^,^^4^. Most of the responding surgeons complied with these recommendations, but the consequences of these strategies varied. For instance, not reaching target(s) did not affect the decision whether to operate for many surgeons. The strict preoperative weight loss regimen more rigorously adopted by American surgeons might be explained by a higher prevalence of obesity in the USA than in Europe^14^. Legal consequences that follow complications may also be an extra incentive to improve outcome by American surgeons. Less experienced surgeons were more strict about preoperative optimization, perhaps reflecting greater reliance on guidelines or the emphasis in surgical training programmes to prevention of complications through preoperative patient optimization^5^^,^^15–17^.

Various bodies have recommended antibiotic prophylaxis administered 1 h before surgery when and if a prosthesis or implant is placed^18^^,^^19^. In this survey, 71.3 per cent surgeons administered antibiotics at least 1 h before surgery. Compliance with protocols and guidelines remains a challenge. Lack of familiarity with guidelines, absence of a hospital infection control team, and reluctance to change practice are recognized factors that contribute to ineffective antibiotic administration^20^.

The existing surgical literature does not provide clear guidance on how to treat individual patients when considering co-morbidity, size of the hernia defect, location of the hernia, and associated loss of domain^1^. The case vignettes confirmed this, as there was no agreement on whether open or laparoscopic hernia repair was preferred in a co-morbid patient. Current guidelines^4^ recommend laparoscopic repair as being safe and effective, with lower risks of wound infection and shorter hospital stay than open repair. American surgeons favoured laparoscopic repair compared with European surgeons, possibly reflecting differences in insurance reimbursement, patient preferences or industry-driven marketing. Open ventral hernia repair has been associated with higher costs than laparoscopic ventral hernia repair in North America^21^.

There was good consensus regarding retrorectus positioning of mesh in open clean ventral incisional hernia repair, but if the rectus muscles could not be approximated, surgeons with less experience more often favoured the posterior component separation technique^22^, whereas experienced surgeons preferred the anterior component separation technique^23^. Newer techniques, such as posterior component separation with transversus abdominis release^24^^,^^25^, had not been reported at the time of the survey.

Analysis of data regarding the choice of mesh showed marked variation with respect to preference for biological meshes. In either primary contaminated, clean recurrent or contaminated recurrent repair, American surgeons chose a biological mesh significantly more often than European surgeons. Guidelines recommend synthetic mesh in clean ventral hernia repair and biological mesh in contaminated ventral hernia repair, but no recommendations exist for clean recurrent or contaminated recurrent repairs^2^^,^^4^ There is no evidence to support the use of biological mesh in these specific circumstances. Current literature in a contaminated environment does not support an obvious advantage for biological over synthetic mesh^26^. A comparative analysis^27^ of similar cohorts in contaminated wound conditions showed that synthetic mesh in a sublay position resulted in significantly less wound morbidity and fewer recurrences compared with biological mesh. Reinforcement with either synthetic or biological mesh during abdominal wall reconstruction with concomitant contaminated procedures, such as stoma reversal, is said to be safe with few mesh removals, but with a higher recurrence rate of up to 30 per cent when using a biological mesh^7^^,^^27–30^. The question remains whether reinforcement with biological mesh in a contaminated environment is expedient and cost-effective.

The majority of surgeons agreed that percutaneous drainage combined with antibiotic treatment was the first step for mesh infection (abscess), independent of the type of repair or mesh used. As for the next step, American surgeons more often proposed a more aggressive approach of mesh removal, whereas European surgeons often continued conservative management with antibiotics. The same was found when comparing more with less experienced surgeons, as well as high- *versus* low-volume surgeons. Surgeons who treat more mesh infections may be more likely to employ an invasive treatment, as they have been confronted more often with its consequences. Current evidence does not support either approach, or give guidance on how to manage mesh infections. Guideline recommendations on mesh infection (and/or abscess) remain based on limited data and expert opinion. In patients without sepsis, it is advised to start with conservative treatment with targeted antibiotic therapy and, if possible, local percutaneous drainage^4^. If this is unsuccessful, mesh removal is necessary. Yet, guidelines do not specify when to explant the mesh and how to repair the defect subsequently. The survey has confirmed the lack of consensus and the need for well designed studies to resolve these issues. As increased surgical volume is associated with better surgical outcomes and lower postoperative complication rates^31^, this is an important consideration in the development of future research protocols.

The results regarding unplanned enterotomy during a laparoscopic repair indicated that most would repair the enterotomy laparoscopically, in line with expert opinion stated by Silecchia et al.: ‘if an enterotomy is recognized without spillage or contamination, a prosthetic repair can be accomplished after closing the enterotomy laparoscopically or open’^4^^,^^32^^,^^33^. It is unknown why some surgeons chose to delay hernia repair, but this may be based on how the amount of spillage was judged. No scientific data exist regarding the number of days or weeks to delay the operation^32^^,^^33^.

This study has limitations. Participants were drawn from professional associations involved in hernia surgery. The authors have no information on the proportion of complex ventral hernia surgery undertaken by surgeons in general. The response rate could not be determined accurately, and was an estimate of the proportion of conference attendees who completed the survey. The proportion of active hernia surgeons on the member list of societies approached was also unknown. As there was no definition for an expert, this level for a ventral hernia surgeon was based arbitrarily on limited data supporting the chosen threshold^34^. The survey did not explore reasons for deviation from guidelines and current evidence, to limit the length of the survey. Existing guidelines relating to mesh infection and contaminated ventral hernia repair are based on limited data and expert opinion. Data on what drives surgeons either to follow or to deviate from guidelines are sparse, and the motivation to do so probably involves many factors. Surgeons who are members of national or international societies and attend meetings are possibly more likely to follow guidelines, and those involved in their development may be more likely to convey their views to colleagues and trainees. The survey questions did not connect experience with annual volume or country of residence, although these were likely to be related. Finally, specific patient characteristics such as immunosuppression or diabetes were not explored in detail regarding choice of mesh, although they may have influenced decision-making.

Based on the findings from this survey, there is a clear need to conduct prospective research that can establish the value of preoperative optimization programmes. Better adherence to guidelines, particularly where evidence exists, such as antibiotic administration, with improved ways of data-sharing must be promoted^35^. Laboratory research into the susceptibility of different meshes to infection and ability to clear infection needs to be undertaken if high-quality clinical trials are to be performed. Critical appraisal of biological meshes in ventral hernia repair requires the performance of RCTs to compare these with synthetic permanent and bioabsorbable meshes in clean and contaminated environments. An international database to collect information on mesh infection. to evaluate and analyse the results of different meshes and therapeutic approaches, would help while trial results are awaited.

##  

*Disclosure*. The authors declare no conflict of interest.

## Supplementary material

Supplementary material is available at *BJS Open* online.

## Supplementary Material

zraa057_Supplementary_Data
