# Supplementary material for: Management of complex ventral hernias: results of an international survey
Source: BJS Open. 2021 Feb 11;5(1):zraa057. doi: 10.1093/bjsopen/zraa057 (PMC7893472; doi:10.1093/bjsopen/zraa057)
Supplement: zraa057_Supplementary_Data [file zraa057_supplementary_data.pdf]

Survey

Please complete the survey below.

Thank you!

Ventral Hernia Infection Survey

Sex
- ◯ Male
- ◯ Female

Age
- ◯ ≤ 30
- ◯ 31-35
- ◯ 36-45
- ◯ 46-55
- ◯ 56-65
- ◯ >65

How many years have you been in practice?
- ◯ ≤ 5
- ◯ 5-10
- ◯ 10-15
- ◯ 15-20
- ◯ >20

Where is your primary practice located?
- ◯ Austria
- ◯ Belarus
- ◯ Belgium
- ◯ Bulgaria
- ◯ Canada
- ◯ Croatia
- ◯ Czech Republic
- ◯ Denmark
- ◯ Finland
- ◯ France
- ◯ Germany
- ◯ Greece
- ◯ Hungary
- ◯ Iceland
- ◯ Ireland
- ◯ Israel
- ◯ Italy
- ◯ Netherlands
- ◯ Norway
- ◯ Poland
- ◯ Portugal
- ◯ Romania
- ◯ Russia
- ◯ Serbia
- ◯ Slovakia
- ◯ Spain
- ◯ Sweden
- ◯ Switzerland
- ◯ Turkey
- ◯ Ukraine
- ◯ United Kingdom
- ◯ United States
- ◯ Other

List the location of your practice. ______________________________

Do you perform open ventral hernia repair?

- ◯ Yes
- ◯ No

Which of the following best estimates your annual volume of open ventral hernia repairs?

- ◯ ≤ 25
- ◯ 25-50
- ◯ 50-100
- ◯ > 100

Do you perform laparoscopic ventral hernia repair?

- ◯ Yes
- ◯ No

Which of the following best estimates your annual volume of laparoscopic ventral hernia repairs?

- ◯ ≤ 25
- ◯ 25-50
- ◯ 50-100
- ◯ > 100

How do you dose prophylactic antibiotics when planning to use permanent mesh for open ventral incisional hernia repair?

- ◯ Preoperative dose at least one hour prior to incision followed by redosing during the case if appropriate
- ◯ Single preoperative dose followed by redosing over the next 24 hours
- ◯ Single preoperative dose followed by redosing > 24 hours
- ◯ I do not use prophylactic antibiotics during cases for ventral incisional hernia repair

If closed-suction drains are placed during the case, do you typically continue antibiotics until they are removed?

- ◯ Yes
- ◯ No

In your estimation, what percentage of all patients who experience ventral hernia recurrence undergo repeat ventral hernia repair?

- ◯ 0-10%
- ◯ 11-20%
- ◯ 21-30%
- ◯ 31-40%
- ◯ 41-50%
- ◯ 51-60%
- ◯ 61-70%
- ◯ 71-80%
- ◯ 81-90%
- ◯ 91-100%

Question A.
Which type of mesh do you prefer to use in the extraperitoneal space when reinforcing the open repair of a > 6 cm ventral incisional hernia at a contaminated surgical site?

- ◯ Permanent synthetic mesh
- ◯ Absorbable synthetic mesh
- ◯ Biologic mesh
- ◯ Primary suture repair with possible autologous tissue transfer, no mesh reinforcement

Which of the following best describes the pore size of the permanent synthetic mesh you would use in this circumstance?

- ◯ Microporous mesh
- ◯ Macroporous mesh
- ◯ Mesh pore size is not a factor in my choice of mesh

Which of the following best describes the density of the permanent synthetic mesh you would use in this circumstance?

- ◯ Lightweight mesh
- ◯ Heavyweight mesh
- ◯ Mesh density is not a factor in my choice of mesh

Following an open ventral incisional hernia repair with extraperitoneal mesh reinforcement at a contaminated surgical site using the technique you specified in question A, the patient experiences a ventral hernia recurrence. If the surgical site is now CLEAN (NO LONGER CONTAMINATED), which type of mesh do you prefer to use in the extraperitoneal space when reinforcing the open repair of the > 6 cm ventral hernia recurrence?

- ◯ Permanent synthetic mesh
- ◯ Absorbable synthetic mesh
- ◯ Biologic mesh
- ◯ Primary suture repair with possible autologous tissue transfer, no mesh reinforcement

Which of the following best describes the pore size of the permanent synthetic mesh you would use in this circumstance?

- ◯ Microporous mesh
- ◯ Macroporous mesh
- ◯ Mesh pore size is not a factor in my choice of mesh

Which of the following best describes the density of the permanent synthetic mesh you would use in this circumstance?

- ◯ Lightweight mesh
- ◯ Heavyweight mesh
- ◯ Mesh density is not a factor in my choice of mesh

Following an open ventral incisional hernia repair with extraperitoneal mesh reinforcement at a contaminated surgical site using the technique you specified in question A, the patient experiences a ventral hernia recurrence. If the surgical site is STILL CONTAMINATED, which type of mesh do you prefer to use in the extraperitoneal space when reinforcing the open repair of the > 6 cm ventral hernia recurrence?

- ◯ Permanent synthetic mesh
- ◯ Absorbable synthetic mesh
- ◯ Biologic mesh
- ◯ Primary suture repair with possible autologous tissue transfer, no mesh reinforcement

Which of the following best describes the pore size of the permanent synthetic mesh you would use in this circumstance?

- ◯ Microporous mesh
- ◯ Macroporous mesh
- ◯ Mesh pore size is not a factor in my choice of mesh

Which of the following best describes the density of the permanent synthetic mesh you would use in this circumstance?

- ◯ Lightweight mesh
- ◯ Heavyweight mesh
- ◯ Mesh density is not a factor in my choice of mesh

After primary/suture repair of a ventral incisional hernia that is > 6cm, most patients develop a recurrence within 10 years. What is the 10 year recurrence rate if an underlay mesh is used to reinforce the repair?

- ◯ 16%
- ◯ 24%
- ◯ 32%
- ◯ 41%

A co-morbid patient (i.e. smoker, obese, diabetic, immunosuppressed, or COPD) with an increased theoretical risk of surgical-site infection has a ventral incisional hernia > 6cm. There is no evidence of wound contamination or active infection. How would you approach this patient?

- ◯ Primary closure without mesh
- ◯ Open repair with permanent synthetic mesh
- ◯ Open repair with absorbable synthetic mesh
- ◯ Open repair with biologic mesh
- ◯ Laparoscopic ventral hernia repair
- ◯ Other

Describe your other approach.

____

Three weeks after open component separation and retrorectus placement of a biologic mesh, a patient develops fever, leukocytosis, and a fluid collection adjacent to the mesh. The patient has no other obvious source of infection. How would you manage this patient? Begin empiric antibiotics and...

- ◯ Observation
- ◯ Place a CT-guided percutaneous drain
- ◯ Open exploration with fluid drainage and complete mesh removal
- ◯ Open exploration with fluid drainage and excision of unincorporated mesh
- ◯ Open exploration with fluid drainage, leaving the mesh in place
- ◯ Other

Describe how you would manage this patient.

____

Three weeks after placement of a permanent-barrier composite mesh during laparoscopic repair, a patient develops fever, leukocytosis, and a fluid collection between the mesh and abdominal wall. The patient has no other obvious source of infection. How would you manage this patient? Begin empirical antibiotics and...

- ◯ Observation
- ◯ Place a CT-guided percutaneous drain
- ◯ Laparoscopic exploration with drainage of fluid and complete mesh removal
- ◯ Open drainage of the fluid and completely excise the mesh
- ◯ Open drainage of the fluid and excise unincorporated mesh
- ◯ Open drainage of the fluid and leave the mesh in place
- ◯ Other

Describe how you would manage this patient.

____

In the scenario above, fluid culture from a CT-guided percutaneous drain reveals MRSA. How would you manage this patient?

- ◯ Continue antibiotics and leave the CT-guided drain in place until resolution of the abscess
- ◯ Open exploration, mesh explantation, and primary hernia repair
- ◯ Open exploration, mesh explantation, and repair with biologic mesh
- ◯ Open exploration, mesh explantation, and repair with absorbable synthetic mesh
- ◯ Laparoscopic removal of the mesh
- ◯ Other

Describe how you would manage this patient.

____

A patient undergoes open ventral hernia repair for a recurrent, incarcerated ventral hernia. A section of small bowel requires resection due to dense adhesions to a prosthetic mesh. There is no inflammation or purulence around the mesh or bowel. What is the surgical wound classification?

- ◯ Clean Wound (Class I)
- ◯ Clean-Contaminated Wound (Class II)
- ◯ Contaminated Wound (Class III)
- ◯ Dirty or Infected Wound (Class IV)

How would you approach a patient with a recurrent parastomal hernia and an infected synthetic mesh without systemic sepsis? Mesh explantation and...

- ◯ Primary closure without mesh
- ◯ Repair with permanent synthetic mesh
- ◯ Repair with absorbable synthetic mesh
- ◯ Repair with biologic mesh
- ◯ Other

Describe your approach.

____

Do you ask patients to stop smoking before ventral incisional hernia repair?

- ◯ Yes
- ◯ No

If yes, how many weeks/months prior to surgery do you ask them to stop smoking? At least...

- ◯ One week
- ◯ Two weeks
- ◯ One month
- ◯ Three months
- ◯ No preference

Do you check urine nicotine and metabolites before surgery to verify smoking cessation?

- ◯ Yes
- ◯ No

If yes, do you require negative results before surgery?

- ◯ Yes
- ◯ No

Do you start morbidly obese patients (BMI > 40kg/m2) on a weight loss regimen before ventral incisional hernia repair?

- ◯ Yes
- ◯ No

If yes, what is your target weight loss prior to surgery?

- ◯ ≤ 5% total body weight
- ◯ 6-10% total body weight
- ◯ 11-15% total body weight
- ◯ 16-20% total body weight
- ◯ >20% total body weight
- ◯ No preference
- ◯ Refer for bariatric surgery

If you establish a target weight loss, will you operate on patients that do not reach the target weight loss?

- ◯ Yes
- ◯ No

Severity grading scales for post-operative complications are important for reporting outcomes. Consider this scenario: A patient develops a post-operative ileus after open ventral hernia repair and requires a short period of total parenteral nutrition (TPN). His recovery is otherwise uneventful. According to all the published grading systems for post-operative complications, what is the correct classification?

- ◯ Grade 0/Normal
- ◯ Grade 1/Mild
- ◯ Grade 2/Moderate
- ◯ ≥ Grade 3/Severe

During an open ventral incisional hernia repair a 4cm gap between the rectus muscles can be easily approximated. The patient has no co-morbidities, no history of wound infection, and no evidence of contamination. How would you proceed?

- ◯ Bridge the defect with mesh
- ◯ Close the fascia primarily without mesh
- ◯ Reinforce the fascial closure with an intraperitoneal mesh
- ◯ Reinforce the fascial closure with a retrorectus mesh (i.e. Rives-Stoppa repair)
- ◯ Reinforce the fascial closure with an onlay mesh
- ◯ Other

Describe your approach.

____

In the patient above, if a 7cm gap between the rectus muscles cannot be approximated without undue tension during an open case. How would you perform the repair?

- ◯ Bridge the defect with mesh
- ◯ Anterior component separation and close the fascia primarily without mesh
- ◯ Anterior component separation and reinforce the fascial closure with an intraperitoneal mesh
- ◯ Anterior component separation and reinforce the fascial closure with a retrorectus mesh (i.e. Rives-Stoppa repair)
- ◯ Anterior component separation and reinforce the fascial closure with an onlay mesh
- ◯ Posterior component separation and reinforce the fascial closure with a rectrorectus mesh
- ◯ Other

Describe your approach.

____

How would you approach a patient with a history of MRSA wound infection and a ventral incisional hernia > 6cm? There is no active infection at this time.

- ◯ Primary closure without mesh
- ◯ Open repair with permanent synthetic mesh
- ◯ Open repair with absorbable synthetic mesh
- ◯ Open repair with biologic mesh
- ◯ Laparoscopic ventral hernia repair
- ◯ Other

Describe your approach.

____

According to the best available evidence, which of following co-morbidities is an independent risk factor for RECURRENCE after ventral hernia repair regardless of technique?

- ◯ Abdominal aortic aneurysm
- ◯ Patient age
- ◯ Steroid use
- ◯ Prostatism
- ◯ Tobacco use

According to the Rives-Stoppa technique for incisional hernia repair, prosthetic mesh is placed in what anatomic plane?

- ◯ Intraperitoneal
- ◯ Preperitoneal
- ◯ Retrorectus
- ◯ Prefascial

Three weeks after open component separation and retrorectus placement of a macroporous mesh, a patient develops fever, leukocytosis, and a fluid collection adjacent to the mesh. The patient has no other obvious source of infection. How would you manage this patient? Begin empiric antibiotics and...

- ◯ Observation
- ◯ Place a CT-guided percutaneous drain
- ◯ Open exploration with fluid drainage and complete mesh removal
- ◯ Open exploration with fluid drainage and excision of unincorporated mesh
- ◯ Open exploration with fluid drainage, leaving the mesh in place
- ◯ Other

Describe how you would manage this patient.

____

If the wound was opened in the scenario above, how would you address the skin closure if there is minimal cellulitis and it can be easily approximated?

- ◯ Primary closure of the skin
- ◯ Loose closure of the skin with draining wicks
- ◯ Pack the wound with gauze and allow closure by secondary intention
- ◯ Apply a negative pressure dressing and allow closure by secondary intention
- ◯ Delayed primary closure
- ◯ Other

Describe your approach.

____

According to the component separation method for closure of abdominal wall defects described by Ramirez et al, what is the typical maximum distance of bilateral advancement in the mid-abdomen?

- ◯ 6cm
- ◯ 10cm
- ◯ 20cm
- ◯ 24cm

How would you approach a patient with a nearby stoma and a ventral incisional hernia > 6cm?

- ◯ Primary closure without mesh
- ◯ Open repair with permanent synthetic mesh
- ◯ Open repair with absorbable synthetic mesh
- ◯ Open repair with biologic mesh
- ◯ Laparoscopic ventral hernia repair
- ◯ Other

Describe your approach.

____

When a midline incision is closed with a continuous monofilament suture, what technique is associated with an increased rate of surgical site infection?

- ◯ Suture length to wound length ratio of < 4:1
- ◯ Suture length to wound length ratio of ≥ 4:1

In diabetic patients, do you check HbA1C before ventral incisional hernia repair?

- ◯ Yes
- ◯ No

If yes, what is your target HbA1C prior to surgery?

- ◯ ≤ 6.0
- ◯ 6.1 - 7.0
- ◯ 7.1 - 8.0
- ◯ 8.1 - 9.0
- ◯ > 9.0
- ◯ No preference

If you establish a target HbA1C, will you operate on patients that do not reach the target HbA1C?

- ◯ Yes
- ◯ No

Within 4 weeks of open ventral hernia repair, up to 21.9% of patients develop a surgical site infection. What is the surgical site infection rate within 4 weeks after laparoscopic ventral hernia repair?

- ◯ 1.5%
- ◯ 2.8%
- ◯ 3.7%
- ◯ 5.1%

In the event of an unplanned enterotomy with minimal spillage during elective laparoscopic ventral incisional hernia repair, how would you typically repair the enterotomy?

- ◯ Repair the enterotomy laparoscopically
- ◯ Convert to open and repair the enterotomy
- ◯ Other

Describe your approach.

____

In the scenario above, how would you address the hernia repair?

- ◯ Delay the hernia repair to a later date
- ◯ Convert to open
- ◯ Remain laparoscopic
- ◯ Other

In the scenario above, which type of mesh would you utilize for the open repair?

- ◯ Biologic mesh
- ◯ Absorbable synthetic mesh
- ◯ Permanent synthetic mesh

In the scenario above, which type of mesh would you utilize for the laparoscopic repair?

- ◯ Biologic mesh
- ◯ Absorbable synthetic mesh
- ◯ Permanent synthetic mesh

Describe your approach.

____

In the scenario above, if you delayed the repair, how long would you wait? At least...

- ◯ 3 days
- ◯ 2 weeks
- ◯ 1 month
- ◯ 6 weeks
- ◯ 3 months
- ◯ 6 months
